# Assessing Communication in Children with Complex Communication Needs: Development of the Italian IVCAA Structured Interview

**DOI:** 10.3390/children13081051

**Published:** 2026-08-06

**Authors:** Sara Rinaldi, Stefania Gazzola, Claudia Maggiulli, Sara Visentin, Elisabetta Cane, Sara Scotto, Daniela Sarti, Luca Andreoli, Elisa Granocchio

**Affiliations:** 1 AULSS 6 Euganea, UOC Infanzia Adolescenza Famiglia e Consultori, 35131 Padova, Italy; sara.rinaldi@aulss6.veneto.it (S.R.); sara.visentin@aulss6.veneto.it (S.V.); 2 Department of Pediatric Neuroscience, Fondazione IRCCS Istituto Neurologico “Carlo Besta”, 20133 Milan, Italy; claudia.maggiulli@istituto-besta.it (C.M.); daniela.sarti@istituto-besta.it (D.S.); luca.andreoli@istituto-besta.it (L.A.); elisa.granocchio@istituto-besta.it (E.G.); 3 Department of Education Sciences, University of Genoa, 16128 Genoa, Italy; 4 Neuropsichiatria Infantile CDR, 10141 Torino, Italy; elisabetta.cane@aslcittaditorino.it (E.C.); sara.scotto@aslcittaditorino.it (S.S.); 5 Department of Humanities and Life Sciences, University School for Advanced Studies IUSS, 27100 Pavia, Italy

**Keywords:** augmentative and alternative communication, communication profile, complex communication needs, developmental disabilities, structured clinical interview

## Abstract

**Background**: Individuals with complex communication needs (CCNs) require comprehensive communication profiles to guide effective interventions, yet the clinical literature offers limited tools to support this process. To address this gap, the “Italian CP & Language Network”—a multidisciplinary group of speech–language pathologists, psychologists, and child neuropsychiatrists—developed a novel, comprehensive clinical instrument specifically designed to evaluate the communication skills of children and adolescents with CCNs. **Methods**: The development process was informed by an initial preliminary survey conducted across participating clinical centres to map out the observation tools routinely used in daily practice. **Results**: Based on these insights, the network designed a structured clinical interview named the Italian IVCAA (Intervista sulla Valutazione delle Competenze e delle Abilità Comunicative). The instrument was structured to systematically assess four core areas: core communication abilities, communicative behaviours, communicative functions, and communicative modalities (vocal, gestural, graphic/symbolic, and written). The Italian IVCAA provides a structured framework that successfully describes an individual’s unique communication profile. It maps out specific strengths and weaknesses across diverse modalities, establishing a standardized baseline for clinical use. **Conclusions:** The Italian IVCAA serves as a valuable clinical tool for identifying communication profiles in individuals with CCNs. It offers a practical starting point for setting tailored goals in augmentative and alternative communication (AAC) interventions and establishes a reliable framework for monitoring changes in communicative competence over time.

## 1. Introduction

Augmentative and Alternative Communication (AAC) is grounded on the recognition of communication as a primary human need [1,2]. AAC aims to support and enhance communication and participation in individuals with complex communication needs (CCNs) [3]. Effective communication is essential for personal development, social participation, and self-care, as well as education and employment [4].

The term “augmentative” highlights that AAC seeks to strengthen and expand existing communication abilities, whatever they may be, rather than replace them. The term “alternative” refers to the use of substitute communication modalities when spoken language is insufficient or absent. For children and adolescents with severely limited or poorly intelligible spoken language, the use of alternative communication modes—alone or in combination (e.g., gestures, images, symbols)—is fundamental for increasing communicative competence and opportunities for participation across everyday settings [3]. From the perspective of the International Classification of Functioning, Disability and Health [5], the effectiveness of habilitation and rehabilitation interventions in communication should be evaluated in terms of the individual’s level of participation within their environment [6].

Assessment of communication and participation needs across daily-life contexts (e.g., home, school) and with different caregivers, as proposed in the Participation Model [4], represents a prerequisite for planning AAC interventions. Such interventions are individualized and cantered on the person, their family and community and must be continually adapted to emerging communication and participation needs in new life contexts (e.g., passage from kindergarten into primary school). In Italy, more than 0.5% of the paediatric population presents complex communication disabilities, which in the absence of appropriate intervention, significantly interfere with cognitive and relational development [7,8,9].

An AAC approach is recommended as early as possible whenever absent or severely limited speech and language development can be expected [10,11,12,13], including in children with receptive language deficits, cognitive impairment, or socio-emotional difficulties.

The literature indicates that there are no minimum thresholds for cognitive development, motor skills, or chronological age below which AAC is contraindicated [14]. Evidence suggests, for example, that children with cerebral palsy (CP) who show delays in language development should be exposed to AAC tools and gestures/signs as early as possible [9,15], even before four years of age [16,17].

Assessment of an individual’s communicative profile requires identifying not only the functions they express but also the level of intentionality and symbolic representation through which these functions are conveyed. In line with the developmental taxonomy defined by Sigafoos et al. [18], communicative acts can be categorized into three distinct levels of intentionality and symbolic complexity. Unintentional acts consist of involuntary or reflex behaviours—such as distress crying or postural shifts—that lack deliberate communicative purpose and acquire meaning solely through caregiver interpretation. Presymbolic intentional acts represent a crucial transition where the individual purposefully directs behaviours toward an interlocutor using non-verbal modalities (e.g., pointing, gaze alternation, or lead-by-hand gestures) to achieve a communicative goal, though without relying on a formal symbolic code. Finally, symbolic intentional acts reflect the most advanced level, wherein the communicative message is intentionally conveyed through shared, conventional symbols, such as spoken words, written text, or formal AAC symbols.

Various approaches can be used to evaluate communication and participation opportunities in daily environments, including written observation records, video recordings, structured templates, and formalized reports [13]. These methods aim to identify discrepancies between the communicative behaviours and participation patterns of peers as compared to those of the individual with CCNs. In this population, standardized tests of receptive and/or expressive language are often difficult to administer because cognitive, communicative–linguistic, visual, or motor impairments may limit test accessibility. For instance, lexical or grammatical comprehension tasks typically require adequate visual scanning and recognition abilities, as well as the motor skills necessary to indicate a correct response.

In Italian clinical practice, the assessment of communication skills in children with CCNs is primarily based on structured or semi-structured clinical observation in play settings, combined with data gathered by interviewing primary caregivers. Several specific tools exist to support clinicians in identifying a child’s communication profile—some are adapted and available in Italian [19,20,21,22,23,24,25], while others are published in English only or in difficult-to-access sources [26,27,28,29,30]. While each of these instruments offers valuable insights and uniquely contributes to the understanding of complex communication profiles, no single tool captures the full spectrum of assessment needs.

Although these instruments provide valuable information, several challenges remain for clinical practice. Available tools vary considerably in their theoretical framework, age range, target population, and administration procedures. Some focus primarily on communication functions, others on participation, environmental factors, or AAC needs; several are not available in Italian, are difficult to access, or require substantial training and time to administer. Consequently, clinicians often need to combine information from multiple sources to obtain a comprehensive picture of a child’s communication profile. These limitations highlight the need for an accessible, structured, and clinically feasible assessment tool that integrates key domains of communication and participation and can be applied across a broad range of children and adolescents with CCNs.

The aim of the present study was to review and integrate these instruments for the development of a caregiver interview in Italian. This original tool is designed to support clinicians in the assessment of communication skills of children and adolescents with CCNs, regardless of diagnosis, type or severity of communication disorder, or other associated impairments (cognitive, sensory, visual, or motor). By identifying both strengths and vulnerabilities, the tool is intended to assist speech–language pathologists in setting treatment goals and monitoring changes in communicative competence over time.

## 2. Materials and Methods

The Italian “CP & Language Network”, established in 2022, brings together speech–language pathologists, psychologists, and child neuropsychiatrists from multiple centres across Italy to investigate communicative–linguistic and speech abilities in children with CP [31]. Within this framework, clinicians have identified a critical lack of Italian-language tools to support the systematic analysis of communicative behaviours in children and adolescents with CCNs. Although the Network was originally created to address communication issues in CP, its members routinely assess and provide intervention for individuals with a broad range of neurodevelopmental conditions associated with CCNs, including autism spectrum disorder, intellectual disability, genetic syndromes, and multiple disabilities. Consequently, the development of the IVCAA was conceived from the outset as diagnosis-independent and intended for use across different clinical populations presenting with CCNs.

During a preliminary phase (2022–2023), a multidisciplinary working group from the Fondazione IRCCS Istituto Neurologico Carlo Besta (20133, Milan, Italy) developed an internal survey, conducted to investigate the clinical organization, patient demographics, and assessment tools used for communication and speech–language evaluations across 11 Italian specialized centres. The questionnaires were addressed to the clinical leads of the components of the “Italian CP & Language Network” group, who had to collect all the information from the whole team.

Further instruments were identified via manual reference list screening (snowballing) of previously included tools (Table 1). This process enabled the group to map existing methods for assessing communication in individuals with CCNs and to identify their respective strengths and limitations. In 2023, a smaller working subgroup of three centres extracted from these instruments key components that were deemed essential for profiling communicative competence and integrated these into a preliminary draft of a new structured interview. The working group included speech–language pathologists, child neuropsychiatrists, and psychologists with expertise in the assessment and rehabilitation of children and adolescents with complex communication needs. Each expert independently evaluated the items in terms of relevance, clarity, and appropriateness for the target population. A consensus process was subsequently implemented, consisting of iterative rounds of discussion aimed at resolving discrepancies and refining the interview content. During these rounds, items were revised, removed, or added based on group feedback.

The initial version of the interview comprised five sections and 44 items. Following iterative discussions among the members of the multidisciplinary working group, substantial revisions were introduced. The section on basic communication skills was expanded from 9 to 20 items and reorganized into four subsections exploring interaction, comprehension, intentionality, and imitation. The section dedicated to communicative behaviours was refined through the explicit specification of communicative functions to analyse the relationship between communicative forms and functions. An additional section was created to document the frequency of communicative functions across daily contexts. The sections on verbal/oral production and gestural production remained unchanged. The picture-based communication section was revised to allow the estimation of the child’s symbolic repertoire, distinguishing between symbols used spontaneously and those used following prompting. In addition, the clinical history section was expanded to include questions on language development and exposure to AAC interventions and systems. The preliminary version was pilot-tested in 2024 through interviews with the primary caregivers of six children with CP who required AAC to support communication in everyday life. The interview was administered by trained speech–language pathologists. Participants ranged in age from 6 years 9 months to 16 years 9 months and included five males and one female. Regarding CP subtype, one child had spastic CP, three had dyskinetic-dystonic CP, and two had dyskinetic choreoathetoid CP. Caregiver responses were systematically analysed by the working group to identify missing information and potential sources of ambiguity.

Pilot testing further informed the refinement of the interview. In particular, caregivers’ responses highlighted the need to collect additional clinically relevant information, including the presence of visual impairments and a more detailed description of communication partners. Particular attention was devoted to refining Section II by more clearly describing the relationships between communicative behaviours and their underlying functions, highlighted through the inclusion of additional examples. This section proved especially useful for delineating the child’s developmental stage and for guiding the selection of intervention targets.

Finally, for children using picture-based communication, two items were revised by replacing the original Likert scale with quantitative symbol ranges (0–20, 20–100, >100 symbols) to facilitate the estimation of expressive vocabulary size in spontaneous and prompted communication.

Agreement among specialists was achieved through deliberation and negotiation until a final version of the interview guide was established. Based on the consensus process and pilot findings, a definitive version of the interview—Intervista per la Valutazione della Comunicazione Aumentativa Alternativa (IVCAA)—was produced in Italian and is accompanied here by an English version to facilitate international dissemination (Supplementary Materials S2 and S3).

## 3. Results

The IVCAA is a structured clinical interview designed to describe the communication profile of children and adolescents with CCNs.

Modality of administration: the IVCAA is administered by a speech–language pathologist to the child’s primary caregiver and can be supplemented by observations from educators, teachers, and other rehabilitation professionals to capture the child’s communicative competence across daily life contexts. The interview may be administered at any point during rehabilitation and repeated as needed to monitor change over time, without any time limits. Although administration time was not formally evaluated during pilot testing, clinical experience suggests that completion of the interview generally requires approximately 20–40 min, whether conducted in person or via telehealth. The duration may vary depending on the child’s communication profile, as are administered only when the corresponding communicative modalities are used by the child. Target population: children and adolescents with CCN, i.e., individuals with communicative–linguistic impairments who are already using AAC strategies or for whom AAC intervention is being considered. There are no restrictions regarding age or underlying diagnosis.

Structure of the interview: the IVCAA consists of seven content sections, preceded by a brief section collecting clinically relevant background information, including diagnosis, associated motor, visual, hearing, and cognitive impairments, language developmental milestones (first words and word combinations), AAC history and current AAC use, and the child’s primary communication partners.

Sections I–III are mandatory for all participants, whereas Sections IV through VII are completed according to the communication modalities employed by the child. Responses are recorded on a three-point Likert scale reflecting the frequency of a behaviour (2 = often, 1 = sometimes, 0 = never) or, for some items, the amount of use of a given modality (0–20; 20–100; >100 occurrences). Data can be entered into an Excel spreadsheet (provided by the authors) to facilitate scoring and analysis.

### 3.1. Section I—Basic Communication Abilities

Twenty questions explore interaction skills, comprehension, communicative intentionality, and imitation abilities. In particular, items on comprehension assess the child’s ability to understand language across increasingly decontextualized situations. Intentionality questions examine, among other aspects, the ability to respond to binary object or verbal choices. Responses are registered through the three-point frequency scale.

### 3.2. Section II—Communicative Behaviours

Caregivers are asked to describe the child’s communicative behaviours at home and in other significant settings in relation to key communicative functions:

− Attention-seeking;
− Requesting objects/actions;
− Protesting;
− Expressing emotions;
− Requesting information;
− Commenting;
− Narrating personal experiences or more complex content.

Four out of nine questions address attention/object/action requests, which represent the earliest communicative functions to emerge during development. Qualitative analysis classifies behaviours as non-communicative, pre-intentional, intentional pre-symbolic, or intentional symbolic (e.g., referential gestures, words, images, written language). Percentages of each behaviour type can be computed using the provided Excel spreadsheet.

### 3.3. Section III—Frequency of Communicative Functions

In this section, caregivers are asked to indicate how often the child produces behaviours serving each communicative function identified in Section II. Responses use the same three-point frequency scale.

### 3.4. Section IV—Oral/Verbal Production

Six questions assess lexical–syntactic development, intelligibility, and pragmatic use of spoken language. Reports from standardized language tests are recommended (when feasible) to complement the caregiver report. Responses use the same three-point frequency scale.

### 3.5. Section V—Gestural Production

Seven items evaluate the type and quantity of gestures, their accuracy and pragmatic use, as well as the ability of the child to combine them with other gestures or spoken words. Responses use the same three-point frequency scale.

### 3.6. Section VI—Image-Based Communication

This most extensive section (15 items) examines communication through visual symbols. Caregivers are asked to report on the type of visual code used (object-based, photographic, iconic), characteristics of the communication device (support type, image organization), and access methods (selection technique, body part used). The accuracy and pragmatic use of images are also investigated. Additional questions (Nos. 53–54) probe the number of images used spontaneously or on request, whereas subsequent questions explore the ability to combine multiple images and to blend them with other communicative modalities. Finally, the questionnaire investigates the actual access to and use of the communication device in everyday contexts, both by the child and by their communication partners. Responses use the same three-point frequency scale.

### 3.7. Section VII—Written Production

The final section includes seven questions designed to explore the child’s use of reading and writing in everyday life. Responses use the same three-point frequency scale.

## 4. Discussion

The IVCAA was developed within the Italian multicentre “CP & Language Network”, drawing on the combined clinical experience of speech–language pathologists, psychologists, and child neuropsychiatrists. The interview responds to the need for an Italian-language tool capable of profiling the communicative abilities of children and adolescents with CCNs, assessing both natural communication skills and the augmentative or alternative modalities used in everyday life with primary communication partners. Several international instruments pursue similar objectives, but only a minority are available in Italian. Moreover, clinicians expressed the need for a comprehensive tool that accommodates the wide heterogeneity of individuals with CCNs.

Following a critical review of existing measures, the working group identified eight key domains as essential for constructing an exhaustive communicative–linguistic profile. These domains guided the development of the IVCAA, which integrates caregiver interview data with direct clinical observation to provide a detailed and individualized communication profile.

Individuals with CCNs often present with motor, cognitive, perceptual, visual, or auditory impairments that limit the feasibility of standardized direct language testing. Indirect tools such as structured caregiver interviews are therefore indispensable for assessment in this population. The IVCAA questions are formulated in simple, ecological language and refer to everyday situations, enabling caregivers to report on the presence and frequency of specific communicative behaviours.

The IVCAA also supports the active involvement of key communication partners—parents, educators, and support teachers—who play a critical role in AAC interventions. Engaging these partners promotes the creation of communication opportunities, encourages the adoption of interactive strategies, and facilitates the maintenance and generalization of acquired communicative skills across new environments [38,39].

Section I allows the verification of basic communication abilities—early communicative signals such as interaction abilities, motivation to communicate, eye contact, shared attentional focus, and turn-taking—that underlie communicative and symbolic development from birth [40,41,42,43]. Section II provides qualitative information on the symbolic level of communicative behaviours, distinguishing between non-intentional, pre-intentional, intentional non-symbolic (e.g., vocalizations, deictic gestures), and intentional symbolic behaviours (e.g., referential gestures/signs, words, images, written language). According to Sigafoos and colleagues [20], communication is considered intentional when a child demonstrates a clear communicative function, alternates gaze between an object and the partner, awaits a response, and continues the behaviour until the goal of communication is achieved. Pre-intentional communication, by contrast, occurs when the expert partner assigns function or meaning to a behaviour without evidence of the child’s deliberate intent [20], as he/she is not yet able to do so independently. Early AAC interventions in developmental age should primarily aim to support the progression from pre-intentional to intentional non-symbolic and eventually symbolic communication by training partners to attribute meaning to the child’s behaviours and to respond contingently [13]. Section III assesses the frequency of use of communicative functions (already collected in Section II when formulating the caregiver questions), allowing for an evaluation of how well the individual is able to use them in daily life. The communicative functions were initially described in studies on the development of communicative intentionality by Bruner and later elaborated by other researchers [44]. These functions include action/object requests, protests, expression of emotions, information requests (e.g., asking the name of a classmate), comments to express opinions about the environment or others, and narration of personal experiences or educational information. Sections IV–VII focus on the analysis of the individual’s predominant communication mode, allowing the examiner to understand which means are most frequently used, depending on the context (verbal, gestural, pictorial, written production). Understanding which modes are used spontaneously and in which contexts help clinicians and families identify the most functional means of expression for specific communicative functions. For instance, expressing primary needs may be easily achieved through the verbal channel (e.g., a single word) or a gestural channel (e.g., a single gesture), whereas expressing opinions or comments, as often required in educational settings, may require another mode (e.g., written production or picture-based sentences), engaging a more advanced level of symbolic communication.

The interview thus allows a better understanding, together with communication partners, of the most suitable mode for each communicative function and context (home, school, or other environments), enabling the individual to choose one or more input/output modes simultaneously according to the function to be expressed [18,45]. In the context of an AAC-based rehabilitation program, it is important that the individual can select the most appropriate alternative communication mode at any given moment, such as gestures or signs [46], picture-based communication [47,48], or written code [49]. Monitoring learned communicative competencies will always be possible through the interview, which can be administered remotely to evaluate the effectiveness of ad hoc rehabilitation interventions.

Compared to other tools used to describe the communication profile of individuals with CCNs, the IVCAA shares several conceptual features with the Communication Matrix [21] while differing in its overall structure and assessment approach. Whereas the Communication Matrix is primarily organized around communicative functions and developmental levels, the IVCAA adopts an ecological perspective and explores communication through examples and situations drawn from everyday contexts and interactions. The interview combines structured and open-ended questions to gather qualitative and quantitative information about communication across different partners and settings. In addition, its modular structure allows clinicians to complete only the sections relevant to the communicative modalities used by the child (e.g., speech, gestures, graphic symbols, or written language). On the other hand, the Communication Matrix enables graphical visualization of the child’s communicative level through a profile of developmental stages, a feature that is not currently available in the IVCAA and could be considered in future developments.

The present study represents an initial step in the development of the IVCAA and, as such, some limitations should be acknowledged. First, the pilot phase involved a small sample and was primarily intended to examine the feasibility and clinical utility of the interview rather than to establish its psychometric properties. In addition, no standardized language assessments or validated communication measures were administered alongside the IVCAA. Therefore, its validity, reliability, and responsiveness to change cannot yet be determined.

## 5. Conclusions

The IVCAA provides a structured framework for profiling the communicative–linguistic abilities of children and adolescents with CCNs. Although developed by Italian specialists with expertise in AAC for children and adolescents, its structure may support future adaptation for adult populations and for use in other linguistic and cultural contexts.

The IVCAA is designed to highlight communicative strengths rather than deficits, supporting the identification of effective alternative modalities and enabling clinicians to monitor the progression of communication skills over time, as recommended in current guidelines [50]. Early access to AAC interventions is critical for children and their families because timely support fosters the development of language and communication abilities and promotes overall cognitive growth and social participation.

Future research should evaluate the IVCAA in a larger and etiologically more heterogeneous sample of children and adolescents with CCNs. Whenever possible, interview findings should be compared with results obtained from standardized measures of receptive and expressive language. Furthermore, comparisons with established communication assessment tools, such as the Communication Function Classification System (CFCS) and the Communication Matrix, will be important for examining convergent validity. Longitudinal studies will also be necessary to determine the sensitivity of the IVCAA to changes in communicative competence over time and its usefulness for monitoring intervention outcomes.

## Figures and Tables

**Table 1:** Assessment tools for communication.

Assessment Tool	Author, Date	Availability in Italian	Target/Clinical Population	Data Gathering Method	Tool Description
ComFor-2 Forerunners in Communication	[24]	[32]	Individuals with autism, intellectual disability, or severe communication difficulties; mental age 12–60 months	Direct: test	Assesses the level achieved by the individual in terms of comprehension and sense-making
Communication Complexity Scale	[30]	NA	Individuals using pre-intentional, presymbolic, or emerging symbolic communication	Direct: observation grid	Assesses expressive communication from pre-intentional to emerging symbolic levels
Communication Function Classification System (CFCS)	[25]	NA	Individuals with CP	Indirect: five-level classification system	Describes how effectively an individual sends and receives messages in everyday situations
Communication Matrix	[21]	[33]	Children with severe communicative, sensory, motor, or cognitive disabilities	Direct and indirect: clinical observation + caregiver interview	Analyses communicative functions across seven levels, from pre-intentional to conventional symbolic behaviours
Evaluation of spontaneous communication with focus on Blissymbols	[34]	NA	Individuals with CCNs using AAC with symbols	Direct: observation grid	Evaluates the use of symbols and symbolic systems in terms of skill and frequency, with specific reference to Bliss Symbols
INteraction CHecklist for Augmentative Communication (INCH)	[26]	NA	Individuals with CCNs	Direct: clinician-completed checklist	Collects information on communication prerequisites and describes communicative behaviours (linguistic, paralinguistic, kinesic, proxemic, and temporal) within the communicative exchange
Inventory of Potential Communicative Acts (IPCA)	[20]	[35]	Children with developmental and/or motor disabilities	Indirect: caregiver interview	Analyses communicative functions through potential communicative acts and concrete examples
Rilevazione Aree Partecipazione—R.A.P.	[23]	Original version	Individuals with CCNs	Indirect: caregiver-reported observations	Assesses levels of autonomy and participation in various life contexts
Student, Environments, Tasks and Tools (SETT) Framework	[28]	NA	Students with disabilities	Indirect: decision-making tool completed after discussion among teachers, therapists, and parents	Collects information on the student and their environment, as well as tasks to guide selection of appropriate assistive technology and support academic success
The Expression of Communicative Intent: Assessment Guidelines	[19]	[36]	Individuals at developmental age	Indirect: caregiver-reported observations	Evaluates communication prerequisites and communicative behaviours used by the child in different life contexts
Triple C: Checklist of Communication Competencies	[29]	NA	Adolescents and adults with severe and/or multiple disabilities	Indirect: caregiver-completed checklist	Identifies communicative development levels from pre-intentional forms to symbolic communication
Augmentative Communication Assessment Profile (ACAP)	[22]	Italian version by (VCAA) [37]	Non-verbal children with autism spectrum disorder	Direct: test	Guides selection of AAC methods for use in rehabilitation interventions

AAC = Augmentative Alternative Communication; CCNs = Complex Communication Needs.

## Data Availability

The original contributions presented in this study are included in the article/Supplementary Materials. Further inquiries can be directed to the corresponding author.

## Supplementary Materials

The following supporting information can be downloaded at: https://www.mdpi.com/article/10.3390/children13081051/s1; File S1: Table S1: Key correspondences between IVCAA sections and available assessment tools; File S2: Italian version of Intervista per la Valutazione della Comunicazione Aumentativa Alternativa-IVCAA; File S3: English version of IVCAA.

## Abbreviations

The following abbreviations are used in this manuscript:

CP	Cerebral Palsy
AAC	Augmentative and Alternative Communication
CCNs	Complex Communication Needs
IVCAA	Intervista per la Valutazione della Comunicazione Aumentativa Alternativa
