# Supplementary material for: Assessing Communication in Children with Complex Communication Needs: Development of the Italian IVCAA Structured Interview
Source: Children (Basel). 2026 Aug 6;13(8):1051. doi: 10.3390/children13081051 (PMC13511035; doi:10.3390/children13081051)
Supplement: Supplementary file 1 [file children-13-01051-s001.zip › IVCAA_Supplementary_Materials_S1_Tables.pdf]

Supplementary Materials:
Table S1. Key correspondences between IVCAA sections and available assessment tools

IVCAA Section Number	Description	Tool
**0**	Primary communication partners for the individual with CCN	INCH, IPCA, SETT Framework
**1**	Basic communicative skills	Communication Matrix, Evaluation of spontaneous communication with focus on Blissymbols, INCH, R.A.P., ACAP
**2**	Analysis of communicative behaviours: non-communicative, pre-intentional, intentional, pre-symbolic, and symbolic behaviours	ComFor-2, Communication Matrix, Evaluation of spontaneous communication with focus on Blissymbols, INCH, IPCA, The Expression of Communicative Intent, Triple C
**3**	Communicative functions and their frequency: attention requests, action/ object requests, protests, expression of emotions, information requests, comments, and narrative skills	Communication Matrix, INCH, IPCA, The Expression of Communicative Intent, Triple C
**4**	Verbal/oral production	Communication Matrix, INCH, R.A.P., SETT Framework, The Expression of Communicative Intent, Triple C
**5**	Gestural production	Communication Matrix, INCH, SETT Framework, The Expression of Communicative Intent, Triple C, ACAP
**6**	Production through images	ComFor-2, Communication Matrix, Evaluation of spontaneous communication with focus on Blissymbols, SETT Framework, Triple C, ACAP
**7**	Written production	Communication Matrix, R.A.P., SETT Framework, Triple C

CCN = Complex Communication Needs
