# Supplementary material for: Assessing Communication in Children with Complex Communication Needs: Development of the Italian IVCAA Structured Interview
Source: Children (Basel). 2026 Aug 6;13(8):1051. doi: 10.3390/children13081051 (PMC13511035; doi:10.3390/children13081051)
Supplement: Supplementary file 1 [file children-13-01051-s001.zip › IVCAA_Supplementary_Materials_S2.pdf]

INTERVISTA PER LA VALUTAZIONE DELLA COMUNICAZIONE AUMENTATIVA ALTERNATIVA

IVCAA

IDEATA NEL CORSO DEGLI INCONTRI DI FORMAZIONE SULLA COMUNICAZIONE E IL LINGUAGGIO DI BAMBINI CON PARALISI CEREBRALI INFANTILI

INTERVISTA PER LA VALUTAZIONE DELLA COMUNICAZIONE AUMENTATIVA ALTERNATIVA

Nome e Cognome	_____________________	Data della compilazione	_____________________
Compilato da	_____________________	Data di nascita	_____________________

Dati anamnestici
Diagnosi________________________ Deficit motori Deficit visivi Deficit uditivi Deficit cognitivi
Quando il bambino ha detto le prime parole_______ Quando ha eseguito prime associazioni________
Quali sono i suoi principali partner comunicativi___________________________
Quando ha iniziato la CAA_______________________
Attualmente usa la CAA: sempre ☐ solo a casa/scuola☐ saltuariamente☐
Titolo studio materno_________________

Compilare il questionario barrando la risposta che si ritiene opportuna.
Compilare le sezioni 4-5-6-7 solo se le modalità comunicative descritte vengono utilizzate dal/la bambino/a.

Sezione 1: Abilità comunicative di base

INTERAZIONE			
1. Il/la bambino/a dimostra motivazione/interesse nel comunicare?	Spesso	A volte	Non ancora
2. Il/la bambino/a mantiene lo sguardo per qualche secondo verso chi parla (ad esempio durante il gioco/pasti …)?	Spesso	A volte	Non ancora
3. Durante il gioco, il/la bambino/a alterna lo sguardo dal volto dell'adulto all'oggetto e viceversa?	Spesso	A volte	Non ancora
4. Durante l'interazione con l'adulto, il/la bambino/a rispetta il suo turno per giocare/parlare?	Spesso	A volte	Non ancora
**COMPRENSIONE**			
5. Il/la bambino/a si gira se chiamato/a per nome?	Spesso	A volte	Non ancora
6. Il/la bambino/a comprende<br>a. Mimica o comportamento non verbale dell'adulto (espressioni del viso, gesto di indicazione, etc.)?	Spesso	A volte	Non ancora
7. Il/la bambino/a comprende<br>Ordini semplici con oggetto presente associato a un vostro gesto esplicativo?<br>*Es. guarda/tocca/prendi la palla indicandola*	Spesso	A volte	Non ancora
8. Il/la bambino/a comprende<br>Ordini semplici con oggetto presente senza un vostro gesto esplicativo?<br>*Es. guarda/tocca/prendi la palla*	Spesso	A volte	Non ancora
9. Il/la bambino/a comprende<br>Ordini semplici senza oggetto presente con un vostro gesto esplicativo?<br>*Es. guarda/tocca/prendi il telefono (facendo il gesto del telefono all'orecchio)*	Spesso	A volte	Non ancora
10. Il/la bambino/a comprende<br>Ordini semplici senza oggetto presente e senza un vostro gesto esplicativo?<br>*Es. dov'è il papà? (o altre persone o cose non presenti)*	Spesso	A volte	Non ancora

INTERVISTA PER LA VALUTAZIONE DELLA COMUNICAZIONE AUMENTATIVA ALTERNATIVA

INTENZIONALITA'			
11. Il/la bambino/a dà un oggetto all'adulto?	Spesso	A volte	Non ancora
12. Il/la bambino/a indica per qualche secondo il gioco che desidera per chiederlo?	Spesso	A volte	Non ancora
13. il/la bambino/a mostra un oggetto o un'immagine durante la lettura di un libretto?	Spesso	A volte	Non ancora
14. il/la bambino/a sceglie tra due cibi/giochi mostrati dall'adulto?<br>Come lo fa?____	Spesso	E volte	Non ancora
15. il/la bambino/a sceglie tra due cibi/giochi su richiesta verbale dell'adulto (*es. vuoi la mela o lo yogurt? senza gli oggetti*)?<br>Come lo fa?____	Spesso	A volte	Non ancora
16. È possibile distinguere quando la scelta del bambino è casuale da quando rispetta la sua preferenza?<br>Se sì, come?____	Spesso	A volte	Non ancora
17. Il/la bambino/a prende spontaneamente il turno (*iniziativa comunicativa*)?<br>Come lo fa?____	Spesso	A volte	Non ancora

IMITAZIONE			
18. Il/la bambino/a imita l'adulto:<br>nella mimica facciale (fa le smorfie)	Spesso	A volte	Non ancora
19. Il/la bambino/a imita l'adulto:<br>nell'uso della voce (riproduce suoni / sillabe / parole che sente)	Spesso	A volte	Non ancora
20. Il/la bambino/a imita l'adulto:<br>nell'uso dei gesti (indicazione, gesto per "*buono*!", gesto per telefonare, gesto per salutare, etc)?	Spesso	A volte	Non ancora

INTERVISTA PER LA VALUTAZIONE DELLA COMUNICAZIONE AUMENTATIVA ALTERNATIVA

Sezione 2: Analisi dei Comportamenti Comunicativi
Cosa fa il/la bambino/a …

21. Quando vuole richiamare la vostra attenzione? (Richiesta di attenzione)

A casa____

A scuola____

Altro (specificare)____

22. Quando ha fame/sete? (Richiesta di oggetto/azione)

A casa____

A scuola____

Altro (specificare)____

23. Quando vuole il suo gioco preferito? (Richiesta di oggetto/azione)

A casa:____

A scuola:____

Altro (specificare):____

24. Quando vuole un abbraccio/una coccola? (richiesta di oggetto/azione)

A casa____

A scuola____

Altro (specificare):____

25. Quando protesta?

A casa____

A scuola____

Altro (specificare):____

26. Quando prova emozioni come Gioia/Paura/Disagio? (espressione di emozioni)

A casa:____

A scuola:____

Altro (specificare):____

27. Quando vuole sapere qualcosa? (es: sapere il nome di un bambino) (richiesta di informazione)

A casa:____

A scuola:____

Altro (specificare):____

28. Quando vuole esprimere una sua opinione un suo commento su sé o sull'ambiente? (commento)

A casa:____

A scuola:____

Altro (specificare):____

29. Quando deve raccontare o riferire qualcosa (es: esperienze personali o informazioni didattiche)

A casa:____

A scuola:____

Altro (specificare):____

Sezione 3: funzioni comunicative (frequenza)

30. Il/la bambino/a produce delle richieste di attenzione?	Spesso	A volte	Mai
31. Il/la bambino/a produce delle richieste di azioni/oggetto?	Spesso	A volte	Mai
32. Il/la bambino/a produce delle proteste?	Spesso	A volte	Mai
33. Il/la bambino/a comunica le sue emozioni?	Spesso	A volte	Mai
34. Il/la bambino/a produce delle richieste di informazioni?	Spesso	A volte	Mai
35. Il/la bambino/a produce dei commenti?	Spesso	A volte	Mai
36. Il/la bambino/a racconta/riferisce?	Spesso	A volte	Mai

Sezione 4: Produzione verbale/orale

37. Il/la bambino/a produce versi di animali?	Spesso	A volte	Mai
38. Il/la bambino/a riproduce melodie conosciute?	Spesso	A volte	Mai
39. Il/la bambino/a dà risposte verbali:			
a. A domande sì/no, ad esempio "*hai fame*?"	Spesso	A volte	Mai
b. A domande a scelta fra due, ad esempio "*vuoi la mela o il cioccolato*?"	Spesso	A volte	Mai
c. A domande aperte, ad esempio "*cosa vuoi mangiare*?"	Spesso	A volte	Mai
40. Ciò che il/la bambino/a dice viene compreso facilmente?			
a. Dai familiari	Spesso	A volte	Mai
b. Da sconosciuti	Spesso	A volte	Mai
41. Ciò che il/la bambino/a dice è aderente rispetto alla situazione in cui ci si trova?	Spesso	A volte	Mai
42. Il/la bambino/a produce:			
a. singole parole ("pappa" / "nanna")	Spesso	A volte	Mai
b. combinazioni di due o più parole? ("*mamma gioco*" per "mamma voglio il gioco")	Spesso	A volte	Mai
c. frasi semplici ("*la mamma gioca*")	Spesso	A volte	Mai
d. frasi complesse ("*la mamma gioca con la macchina*")	Spesso	A volte	Mai

Note e osservazioni:

Sezione 5: Produzione gestuale

43. Il/la bambino/a usa segni appartenenti alla Lingua dei Segni Italiana (LIS)?	Spesso	A volte	Mai
44. Il/la bambino/a usa gesti personalizzati?	Spesso	A volte	Mai
45. Il gesto/segno viene eseguito accuratamente?	Spesso	A volte	Mai
46. Il/la bambino/a usa:			
a. Gesti/segni singoli	Spesso	A volte	Mai
b. Combinazioni di più gesti/segni	Spesso	A volte	Mai
47. Il gesto/segno che viene prodotto è aderente rispetto alla situazione in cui ci si trova?	Spesso	A volte	Mai
48. Il/la bambino/a accompagna il gesto alla produzione verbale?	Spesso	A volte	Mai
49. Il/la bambino/a usa spontaneamente gesti/segni? Quanti ne usa?________________________	Spesso	A volte	Mai

Note e osservazioni:

INTERVISTA PER LA VALUTAZIONE DELLA COMUNICAZIONE AUMENTATIVA ALTERNATIVA

Sezione 6: Produzione per immagini

50. Per comunicare, il/la bambino/a usa:<br>a. Oggetti<br>b. Fotografie<br>c. Immagini (iconiche)<br>d. Immagini (astratte)<br>e. Altro____________________<br>Specificare: ____	 <br>Spesso<br>Spesso<br>Spesso<br>Spesso	 <br>A volte<br>A volte<br>A volte<br>A volte	 <br>Mai<br>Mai<br>Mai<br>Mai
51. Che tipo di supporto utilizzate?<br>a. Tablet<br>b. Tabella cartacea<br>c. Comunicatori cartacei personalizzati<br>d. Altro____________________	 Spesso<br>Spesso<br>Spesso<br>Spesso	 A volte<br>A volte<br>A volte<br>A volte	 Mai<br>Mai<br>Mai<br>Mai
52.<br>a. Quanto spesso utilizzate una modalità di scelta tra due immagini?<br>b. Quanto spesso utilizzate una organizzazione per categorie (cibi, giochi, azioni…) per sostenere la comunicazione?<br>c. Quanto frequentemente utilizzate altre modalità di organizzazione delle immagini all'interno dei supporti?	 Spesso<br>Spesso<br>Spesso	 A volte<br>A volte<br>A volte	 Mai<br>Mai<br>Mai
53. a) Il/la bambino/a consegna all'adulto l'immagine che ha scelto?<br>b) Il/la bambino/a indica l'immagine che ha scelto?<br>Con quale parte del corpo avviene la scelta fra i target?<br>____	Spesso<br>Spesso	A volte<br>A volte	Mai<br>Mai
54. L'azione per indicare la scelta è eseguita accuratamente?	Spesso	A volte	Mai
55. L'uso dei simboli/immagini/ecc è aderente rispetto alla situazione?	Spesso	A volte	Mai
56. a) Quante immagini utilizza su richiesta a casa?<br>b) Quante immagini utilizza su richiesta a scuola?<br>c) Quante immagini utilizza su richiesta in altri luoghi?	>100<br>>100<br>>100	20-100<br>20-100<br>20-100	0-20<br>0-20<br>0-20
57. a) Quante immagini utilizza spontaneamente a casa?<br>b) Quante immagini utilizza spontaneamente a scuola?<br>c) Quante immagini utilizza spontaneamente in altri luoghi?	>100<br>>100<br>>100	20-100<br>20-100<br>20-100	0-20<br>0-20<br>0-20
58. Il/la bambino/a combina su richiesta più immagini insieme per produrre enunciati più lunghi?	Spesso	A volte	Mai
59. Il/la bambino/a combina spontaneamente più immagini insieme per produrre enunciati più lunghi?	Spesso	A volte	Mai
60. Il/la bambino/a supporta l'indicazione con la produzione vocale/verbale?	Spesso	A volte	Mai
61. Il/la bambino/a cerca spontaneamente il comunicatore/tablet/ecc?	Spesso	A volte	Mai
62. a) Il/la bambino/a utilizza il comunicatore/tablet in ambienti conosciuti e con persone conosciute?<br>b) Il/la bambino/a utilizza il comunicatore/tablet in ambienti nuovi e con persone sconosciute?	Spesso<br>Spesso	A volte<br>A volte	Mai<br>Mai
63. Il/la bambino/a accompagna l'uso dei simboli alla produzione vocale/verbale?	Spesso	A volte	Mai

64. Il/la bambino/a è esposto alla CAA negli ambienti di casa/scuola? (etichettatura, strisce di attività ecc…)	Spesso	A volte	Mai
Note e osservazioni:			

Sezione 7: Produzione scritta

65. Il/la bambino/a usa il tablet/PC per scrivere?	Spesso	A volte	Mai
66. Il/la bambino/a scrive singole parole?	Spesso	A volte	Mai
63. Il/la bambino/a scrive frasi (semplici o complesse)?	Spesso	A volte	Mai
68. Il/la bambino/a scrive dei testi?	Spesso	A volte	Mai
69. Il/la bambino/a legge/riconosce numeri e scritte che incontra nella vita quotidiana (es. numero del tram, insegne…)	Spesso	A volte	Mai
70. Il/la bambino/a legge testi cartacei o su tablet/PC?	Spesso	A volte	Mai
71. Il/la bambino/a ascolta testi con sintesi vocale/audiolibri?	Spesso	A volte	Mai
Note e osservazioni:			

INTERVISTA PER LA VALUTAZIONE DELLA COMUNICAZIONE AUMENTATIVA ALTERNATIVA

COMPORTAMENTI RILEVATI DALLA SEZ. 2

COMPORTAMENTO NON COMUNICATIVO	
Bisogno non espresso	
Anticipazione da parte del caregiver	
COMPORTAMENTO PRETERINTEZIONALE	
Comportamento solitario	
Pianto/capricci	
Mimica facciale/sguardo	
Vocalizzi	
altri comportamenti fisici …	
COMPORTAMENTO INTENZIONALE	
Ricorso all'adulto	
Mimica facciale/sguardo	
Vocalizzi	
altri comportamenti fisici …	
COMPORTAMENTO PRESIMBOLICO	
Indicazione/mano tesa nello spazio	
GESTO DEITTICO (MOSTRARE/DARE)	
gesto deittico + vocalizzo	
COMPORTAMENTO SIMBOLICO	
Indicazione + produzione verbale	
Gesti referenziali	
Gesti referenziali + produzione verbale	
Produzione verbale (onomatopee, parole, frasi)	
PRODUZIONE CON IMMAGINI	
