# Supplementary material for: Assessing Communication in Children with Complex Communication Needs: Development of the Italian IVCAA Structured Interview
Source: Children (Basel). 2026 Aug 6;13(8):1051. doi: 10.3390/children13081051 (PMC13511035; doi:10.3390/children13081051)
Supplement: Supplementary file 1 [file children-13-01051-s001.zip › IVCAA_Supplementary_Materials_S3.pdf]

IVCAA - INTERVIEW FOR THE ASSESSMENT OF AUGMENTATIVE AND ALTERNATIVE COMMUNICATION

Child's name and surname: ______________________

Date of birth: ______________________

Respondent (caregiver): ______________________

Completed by: ______________________

Date of completion: ______________________

Background information

Diagnosis: ____

☐ Motor deficits ☐ Visual deficits ☐ Hearing deficits ☐ Cognitive deficits

Age at first words: ____

Age at first word combinations: ____

Primary communication partners: ____

Age at introduction of AAC: ____

Current AAC use: ☐ Always ☐ Only at home/school ☐ Occasionally

Mother's education level: ____

<u>Please complete the questionnaire by ticking the most appropriate response.</u>

<u>Sections 4–7 should be completed only if the corresponding communication modes are used by the child.</u>

Section 1 – Basic Communication Abilities

INTERACTION

(mark one for each item: **Often / Sometimes / Not yet**)

1. Does the child show motivation or interest in communicating? Often / Sometimes / Not yet
2. Does the child maintain eye contact for a few seconds with the speaker (e.g., during play or mealtimes)? Often / Sometimes / Not yet
3. During play, does the child alternate gaze between the adult's face and the object, and back again? Often / Sometimes / Not yet
4. During interaction with an adult, does the child take turns appropriately when playing or speaking? Often / Sometimes / Not yet

COMPREHENSION

(mark one for each item: **Often / Sometimes / Not yet**)

5. Does the child turn when called by name?

6. Does the child understand facial expressions or nonverbal behaviours of the adult (e.g., pointing gestures, facial cues)?
7. Does the child understand simple commands when the object is present and associated with your explanatory gesture?

   Example: "Look/touch/get the ball" while pointing to the ball.
8. Does the child understand simple commands when the object is present without your explanatory gesture?

Example: "Look/touch/get the ball" without pointing.

9. Does the child understand simple commands when the object is not present but associated with your explanatory gesture?

   Example: "Look/touch/get the phone" while making a phone gesture.

10. Does the child understand simple commands when the object is not present and without your explanatory gesture?

   Example: "Where is Dad?" (or other absent people/objects).

INTENTIONALITY

(mark one for each item: **Often / Sometimes / Not yet**)

11. Does the child give an object to the adult?
12. Does the child point for a few seconds to the toy he/she wants to request?
13. During book reading, does the child show an object or a picture to the adult?
14. Does the child choose between two foods/toys shown by the adult?

    If yes, how does the child make the choice? ______________________

15. Does the child choose between two foods/toys when prompted verbally by the adult (e.g., "Do you want the apple or the yogurt?" without the objects present)?

    If yes, how does the child make the choice? ________________________

16. Is it possible to distinguish when the child's choice is random versus reflecting a true preference?

    If yes, how? ____

17. Does the child spontaneously take a turn (communicative initiative)?

    If yes, how? ____

IMITATION

(mark one for each item: **Often / Sometimes / Not yet**)

18. Does the child imitate the adult in facial expressions (e.g., makes funny faces)?
19. Does the child imitate the adult using the voice (e.g., reproduces sounds/syllables/words that he/she hears)?
20. Does the child imitate the adult using gestures (e.g., pointing, gesture for "good!", telephone gesture, waving…)?

Section 2 – Analysis of Communicative Behaviours

Describe the child's behaviour (mode/context) in situations such as:

21. When the child wants to get your attention (Attention-seeking)

    At home: ____

    At school: ____

    Other (specify): ____

22. When the child is hungry or thirsty (Request for object/action)

    At home: ____

    At school: ____

    Other (specify): ____

23. When the child wants his/her favourite toy (Request for object/action)

At home: ____

At school: ____

Other (specify): _______________________________________

24. When the child wants a hug or cuddle (Request for object/action)

    At home: ____

    At school: ____

    Other (specify): _______________________________________

25. When the child protests

    At home: ____

    At school: ____

    Other (specify): _______________________________________

26. When the child experiences emotions such as joy, fear, or discomfort (Expression of emotions)

    At home: ____

    At school: ____

    Other (specify): _______________________________________

27. When the child wants to know something (e.g., wants to know another child's name) (Request for information)

    At home: ____

    At school: ____

    Other (specify): _______________________________________

28. When the child wants to express an opinion or make a comment about self or the environment (Comment)

    At home: ____

    At school: ____

    Other (specify): _______________________________________

29. When the child needs to tell or report something (e.g., personal experiences or school-related information)

    At home: ____

    At school: ____

    Other (specify): _______________________________________

Section 3 – Communicative Functions

(mark one for each item: **Often / Sometimes / Not yet**)

30. Does the child produce attention-seeking requests?
31. Does the child produce requests for actions or objects?
32. Does the child protest?
33. Does the child communicate emotions?
34. Does the child produce requests for information?

35. Does the child produce comments?
36. Does the child tell or report information?

Section 4 – Verbal/Oral Production

(mark one for each item: **Often / Sometimes / Not yet**)

37. Does the child produce animal sounds?
38. Does the child reproduce familiar melodies?
39. Does the child give verbal answers:

    a. To yes/no questions (e.g., "Are you hungry?")

    b. To forced-choice questions (e.g., "Do you want the apple or the chocolate?")

    c. To open-ended questions (e.g., "What do you want to eat?")

40. Is what the child says easily understood:

    a. By family members

    b. By unfamiliar listeners

41. Is what the child says appropriate to the situation?
42. Does the child produce:

    a. Single words (e.g., "food/bed")

    b. Two-word combinations (e.g., "*Mom toy*" for "Mom, I want the toy")

    c. Simple sentences (e.g., "*Mom plays*")

    d. Complex sentences (e.g., "*Mom plays with the car*")

Notes and observations:

Section 5 – Gestural Production

(mark one for each item: **Often / Sometimes / Not yet**)

43. Does the child use signs from Italian Sign Language (LIS)?
44. Does the child use personalized gestures?
45. Is the gesture/sign performed accurately?
46. Does the child use:

    a. Single gestures/signs

    b. Combinations of multiple gestures/signs

47. Is the gesture/sign appropriate to the situation?
48. Does the child combine the gesture with verbal production?
49. Does the child use gestures/signs spontaneously?

    How many? ______________________

Notes and observations:

Section 6 – Picture-Based Communication

(mark one for each item: **Often / Sometimes / Not yet)**

(for items 52-53 mark **0-20 / 20-100 / > 100**)

50. To communicate, does the child use:

a. Objects
b. Photographs
c. Pictures (iconic)
d. Pictures (abstract)
e. Other: ___________________

Please specify: _______________________________

51. What type of support do you use?

a. Tablet
b. Paper-based chart
c. Personalized paper communicators
d. Other: ___________________

52.

a. How often do you use a choice between two images?
b. How often do you use category organization (e.g., food, toys, actions) to support communication?
c. How often do you use other organization methods for images within the communication supports?

53.

a. Does the child hand the chosen image to the adult?
b. Does the child point to the chosen image?

Which body part is used to select the target?

____

54. Is the action to indicate the choice performed accurately?

55. Is the use of symbols/images appropriate for the situation?

56.

a. How many images does the child use on request at home?
b. How many images does the child use on request at school?
c. How many images does the child use on request in other settings?

57.

a. How many images does the child use spontaneously at home?
b. How many images does the child use spontaneously at school?
c. How many images does the child use spontaneously in other settings?

58. Does the child combine multiple images on request to produce longer utterances?

59. Does the child combine multiple images spontaneously to produce longer utterances?

60. Does the child support the indication with vocal/verbal production?

61. Does the child seek the communicator/tablet/etc. spontaneously?

62.

   a. Does the child use the communicator/tablet in familiar environments and with familiar people?
   b. Does the child use the communicator/tablet in new environments and with unfamiliar people?

63. Does the child accompany the use of symbols with vocal/verbal production?

64. Is the child exposed to AAC in home/school environments? (e.g., labeling, activity strips, etc.)

Notes and observations:

Section 7 – Written Production

(mark one for each item: **Often / Sometimes / Not yet)**

65. Does the child use a tablet/PC for writing?
66. Does the child write single words?
67. Does the child write sentences (simple or complex)?
68. Does the child write longer texts?
69. Does the child read/recognize numbers and written words encountered in daily life (e.g., tram numbers, signs, etc.)?
70. Does the child read texts on paper or on a tablet/PC?
71. Does the child listen to texts via text-to-speech or audiobooks?

Notes and observations:

Coding of Behaviours (from Section 2)

The child's behaviours can be classified as:

Non-communicative behaviour

i. unexpressed needs
ii. anticipation by the caregiver

Pre-intentional behaviour

iii. solitary behaviour
iv. crying/tantrums
v. facial expressions/gaze
vi. vocalisations
vii. other physical behaviours

Intentional behaviour

viii. adult-directed behaviours
ix. facial expressions/gaze
x. vocalisations
xi. other physical behaviours

Pre-symbolic behaviour

xii. pointing/reaching
xiii. deictic gesture (show/give)

xiv. deictic gesture + vocalisation

Symbolic behaviour

xv. pointing + verbal production
xvi. referential gestures
xvii. referential gestures + verbal production
xviii. verbal production (onomatopoeias, words, sentences)
xix. picture-based productions
